# Rational coupled dynamics network manipulation rescues disease-relevant mutant cystic fibrosis transmembrane conductance regulator†
†Electronic supplementary information (ESI) available. See DOI: 10.1039/c4sc01320d



**DOI:** 10.1039/c4sc01320d

**Published:** 2014-11-20

**Authors:** Elizabeth A. Proctor, Pradeep Kota, Andrei A. Aleksandrov, Lihua He, John R. Riordan, Nikolay V. Dokholyan

**Affiliations:** a Curriculum in Bioinformatics and Computational Biology , University of North Carolina , Chapel Hill , NC 27599 , USA . Email: dokh@unc.edu; b Program in Molecular and Cellular Biophysics , University of North Carolina , Chapel Hill , NC 27599 , USA; c Department of Biochemistry and Biophysics , University of North Carolina , Chapel Hill , NC 27599 , USA; d Cystic Fibrosis Treatment and Research Center , University of North Carolina , Chapel Hill , NC 27599 , USA

## Abstract

Many cellular functions necessary for life are tightly regulated by protein allosteric conformational change, and correlated dynamics between protein regions has been found to contribute to the function of proteins not previously considered allosteric. The ability to map and control such dynamic coupling would thus create opportunities for the extension of current therapeutic design strategy. Here, we present an approach to determine the networks of residues involved in the transfer of correlated motion across a protein, and apply our approach to rescue disease-causative mutant cystic fibrosis transmembrane regulator (CFTR) ion channels, ΔF508 and ΔI507, which together constitute over 90% of cystic fibrosis cases. We show that these mutations perturb dynamic coupling within the first nucleotide-binding domain (NBD1), and uncover a critical residue that mediates *trans*-domain coupled dynamics. By rationally designing a mutation to this residue, we improve aberrant dynamics of mutant CFTR as well as enhance surface expression and function of both mutants, demonstrating the rescue of a disease mutation by rational correction of aberrant protein dynamics.

## Abstract 2

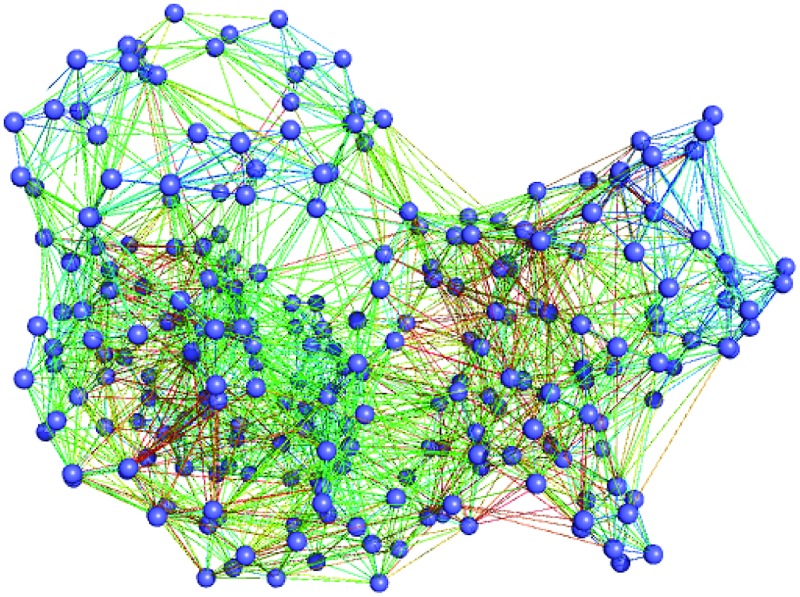
A novel approach identifying networks of residues involved in *trans*-protein dynamic coupling is applied to rescue mutant CFTR.

## Introduction

Protein allostery is a ubiquitous mechanism central to the regulation of many cellular processes, such as enzyme catalysis and signal transduction.^1,2^ An allosteric change involves the protein population ensemble redistributing itself among the available conformations.^2–4^ NMR studies of dynamic coupling between residues in proteins support the idea that allostery is a common intrinsic property of many proteins.^3,5,6^ Exerting control over such dynamic coupling interactions could open the door for novel therapeutic strategies in diseases such as cystic fibrosis. However, predicting and designing the effect of coupling interactions on protein structure and function remains a challenge. Determining interaction networks that couple distant sites of the protein and identifying hub (“hot spot”) residues that control the dynamics of such coupling interactions are essential steps to uncovering the molecular mechanisms of protein allostery.^7^ Previous efforts toward these ends have focused mainly on searching for new allosteric sites and mechanisms; X-ray crystallographic studies of bound and unbound structures have offered particularly important structural insights into allosteric regulation.^7^ However, analysis of the static structures of allosteric end states cannot provide a complete picture of the inter-residue dynamics and interactions involved in allosteric conformational change or the coupling of dynamics at distal sites. NMR studies of protein dynamics have been pivotal in identifying “hidden” networks of residues with strong dynamic coupling.^6,8^ Thermodynamic mutation cycles,^9^ which measure the coupling between two mutation sites by their mutual contribution to protein stability, provide a direct method to systematically probe such relations between protein sites. However, due to experimental limitations and practical considerations, a large-scale study using these methods would be prohibitively laborious and time consuming.

Computational methods can be used to probe the coupling of amino acids and to identify networks of residues controlling protein conformational changes. For example, sequence-based approaches can reveal co-evolving residues likely to be energetically or functionally coupled.^10–14^ However, the application of sequence-based approaches, which rely on evolutionary information, is limited by the availability of homologous sequences and complicated by the fact that evolutionary conservation is often driven by factors other than function (*e.g.* stability, folding kinetics).^15,16^

Recently, we have proposed that coupled dynamics plays a critical role in the pathophysiology of cystic fibrosis (CF).^17,18^ Over 1500 mutations in the CFTR gene have been identified in patients with CF. The protein product of this gene, CFTR, plays a fundamental role in epithelial ion transport, providing a rate-limiting step in the regulation of salt secretion and reabsorption. In approximately 90% of CF patients, the deletion of a single phenylalanine (ΔF508) in its first nucleotide-binding domain (NBD1) results in misfolding and misassembly of the protein (ΔF508-CFTR).^17^ Cheng *et al*. determined that the ΔF508 mutation prevents maturation and trafficking of CFTR to the cell membrane,^19^ although maturation and partial function of ΔF508-CFTR have been observed at sub-physiological temperature (<30 °C) in various cell types.^20–23^ Second site mutations, like the commonly used I539T substitution, can improve maturation,^24,25^ but ΔF508-CFTR rescued in this manner exhibits poor function at physiological temperature, indicating that the fundamental defect in ΔF508-CFTR maturation and function is a consequence of reduced thermal stability.^26^ This hypothesis is further supported by the fact that F508 plays a critical role in interfacing NBD1 with the fourth cytoplasmic loop of CFTR, thus contributing significantly to the structural integrity and stability of the protein.^27,28^ Therefore, effective therapeutic rescue of mutant CFTR requires restoration of thermal stability, often mediated by protein fluctuations and dynamics, making mutant CFTR an ideal system for development of methods for dynamic control.

Here, we develop a widely applicable computational methodology that utilizes concepts from graph theory to identify specific residues that propagate dynamic coupling effects between structurally distant sites in proteins. We apply our approach to the rescue of ΔF508-CFTR, using data from discrete molecular dynamics simulations, performable on a personal computer, to identify hot spot sites. We then utilize mutagenesis to demonstrate control over dynamic coupling between two distant regions of NBD1. We show that rational mutagenesis of the identified bottleneck sites dramatically rescues aberrant dynamics, as well as dramatically improving maturation and function of ΔF508-CFTR in mammalian cells. We further demonstrate the ability of our method to identify sites important to wild-type protein function and stability by showing that our designed mutation can also rescue the adjacent but distinct and more severe disease mutant, ΔI507-CFTR.

## Results

### Heightened thermal fluctuations in the regulatory insertion propagate to the SDR and ATP-binding sub-domain

**Fig. 1:**
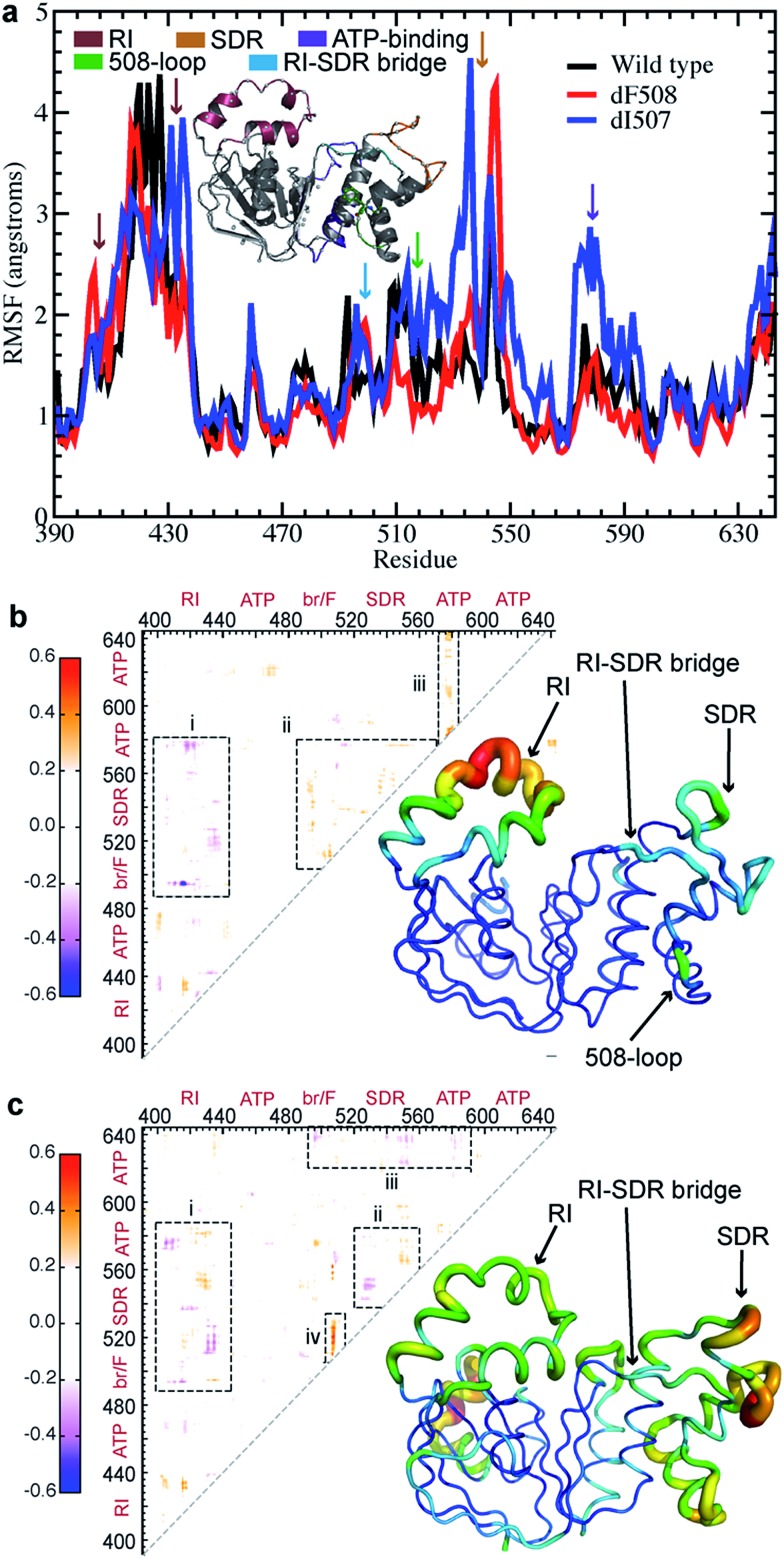
Deletion of F508 or I507 results in altered coupling between regions of NBD1. (a) The root mean square fluctuations (RMSF) over the simulation of each residue in WT NBD1 compared with those in ΔF508- and ΔI507-NBD1 suggests increased flexibility in several regions upon deletion mutation. (b) Difference map derived from the correlation maps of WT and ΔF508 NBD1. Blue denotes lost correlation upon deletion mutation, while red denotes gained correlation. Tube representation of NBD1 protein dynamics highlights changes in fluctuations in NBD1 upon deletion of F508. The thickness and color of the tube represent the extent of change in dynamics of the corresponding region during the simulation. Warmer colors indicate greater increase in flexibility, while colder colors indicate no change. (c) Same as (b), for ΔI507-NBD1.

We and others have reported that the NBD1 regulatory insertion (RI, residues 404-435) influences the dynamics of NBD1 in CFTR,^18,26,29^ but the nature and mechanism of this influence are still unknown. To elucidate the effects of RI within NBD1, we perform discrete molecular dynamics (DMD) simulations^30,31^ of wild type (WT), ΔF508-, and ΔI507-NBD1. We find that thermal fluctuations increase and/or are shifted significantly in several key regions of NBD1 upon deletion of F508 or I507: the RI, structurally diverse region (SDR), residues 532-552,^29^ RI-SDR bridge (residues 492-502), F508-loop (residues 507-514), and part of the ATP-binding sub-domain (residues 570-600) (regions indicated by color-coded arrows, Fig. 1a). These heightened fluctuations suggest the possibility of dynamic coupling between the affected regions, which would transfer a perturbation in one region to any of the others. For example, deletion of F508 or I507 in the F508 loop affects the dynamics of RI, the RI-SDR bridge, the SDR, and the ATP-binding sub-domain.

### F508 deletion results in non-native internal coupling of the α-subdomain and ATP-binding subdomain

In order to confirm our hypothesis of coupled dynamics between key structural regions of NBD1, we perform covariation analysis of dynamic fluctuations observed in simulations.^32^ In ΔF508-NBD1, we find a marked decoupling of the RI from the entire α-subdomain and parts of the ATP-binding subdomain as compared to the wild type (Fig. 1b-i). In addition, we observe an increase in coupling between the various key regions of the α-subdomain and the ATP-binding subdomain (Fig. 1b-ii), and an increase in internal coupling in the ATP-binding subdomain (Fig. 1b-iii). From these findings, we conclude that the F508 loop, the SDR, the RI-SDR bridge, the RI, and the ATP-binding subdomain are inter-connected in a network of coupled dynamics within NBD1. These changes suggest that the deletion of F508 from the F508 loop could cause dynamic instability and increased fluctuations in this region, which lead to decoupling from the RI. The RI decouples from the entire α-subdomain, leading to the gain of non-native coupling within that region. Ultimately, the observed increase in fluctuations and changes to dynamic coupling within NBD1 may result in domain instability and consequent misprocessing of ΔF508-CFTR. Deletion of the RI eliminates this transfer of dynamics through the domain, rescuing the protein as observed.^26^

### I507 deletion results in non-native coupling between the SDR and the ATP-binding sub-domain

Instead of the complete decoupling of the RI from the α-subdomain seen with the F508 deletion (discussed above), ΔI507-NBD1 features a shift in the regions of RI that undergo coupling, losing coupling in one area only to gain it in adjacent residues (Fig. 1c-i). We detect a similar shift of coupling interactions in the SDR, with its coupling to the RI (Fig. 1c-i), and in addition note that the SDR loses coupling within itself (Fig. 1c-ii), likely due to the increase in fluctuations in this area (Fig. 1a). The perturbation to the SDR also includes a gained non-native coupling with part of the ATP-binding subdomain (Fig. 1c-ii), which decouples from the α-subdomain (Fig. 1c-iii). Lastly, we find a strong non-native coupling of the SDR with the F508 loop (Fig. 1c-iv), likely the perturbation caused by the deletion mutation. Combining these findings, we conclude that the deletion of I507 causes a perturbation to coupled dynamics that is transferred by coupled dynamics through the SDR to the RI and the ATP-binding subdomain, shifting coupling interactions all throughout NBD1, which severely affects the dynamic stability of the domain. Likewise, the non-native coupling of the ATP-binding subdomain to the SDR and the loss of internal coupling in the ATP-binding subdomain could affect the binding of ATP, which is stabilizing to NBD1 under normal conditions. In the absence of ATP-binding, NBD1 is significantly destabilized, potentially compounding the effects of the ΔI507 mutation.

### Network formalism of NBD1 dynamics reveals bottleneck residues that control dynamic coupling

In order to deduce the pathways through which these changes in dynamic coupling occur, for each system of wild type, ΔF508, and ΔI507 NBD1, we represent the pairwise correlation coefficients between residues as a complete graph, with the correlation between every possible pair of residues represented as a connection between nodes (an edge). We then weight this graph (G(N,E), where N is the set of nodes and E is the set of edges) such that each edge is enumerated by the correlation coefficient between the corresponding pair of residues. Weighting enables us to isolate those edges having the most significant impact on the transduction of fluctuations across the protein. To identify the specific residues mediating dynamic coupling within NBD1, we impose a correlation cutoff to our graph, eliminating edges with a weight below our cutoff until the largest component of the resulting disconnected sub-graph comprises approximately 50% of the total number of nodes in the graph (Fig. S1†). This cutoff is at the critical threshold, where the network exhibits critical properties^33^ and transitions from connected to disconnected.^34^ Our rationale with this construction is to create an algorithm for network mapping that is without free parameters. From the resulting network, we determine whether a node is critical for the connectivity of the largest component in the sub-graph by monitoring the topological changes in the sub-graph as we iteratively remove each node from the sub-graph. If removing node *a* splits the largest sub-graph into two or more components, then node *a* is a critical node, or “bottleneck,” in the network. A bottleneck residue is a residue with the ability to affect the entire network because of its positioning as the lone connection between at least two regions of a network (*i.e.*, an unavoidable step in the coupling of dynamics between one region and the other). Mutation or modification of a bottleneck residue can affect the dynamics, stability, and allosteric or binding behavior of a protein or domain. We find several such bottleneck residues in the dynamic networks of ΔF508, ΔI507, and wild type NBD1, which we perturb to ameliorate the pathological dynamics of the mutant NBD1s.

### Network bottlenecks control the transfer of heightened thermal fluctuations from the RI of ΔF508-NBD1

**Fig. 2:**
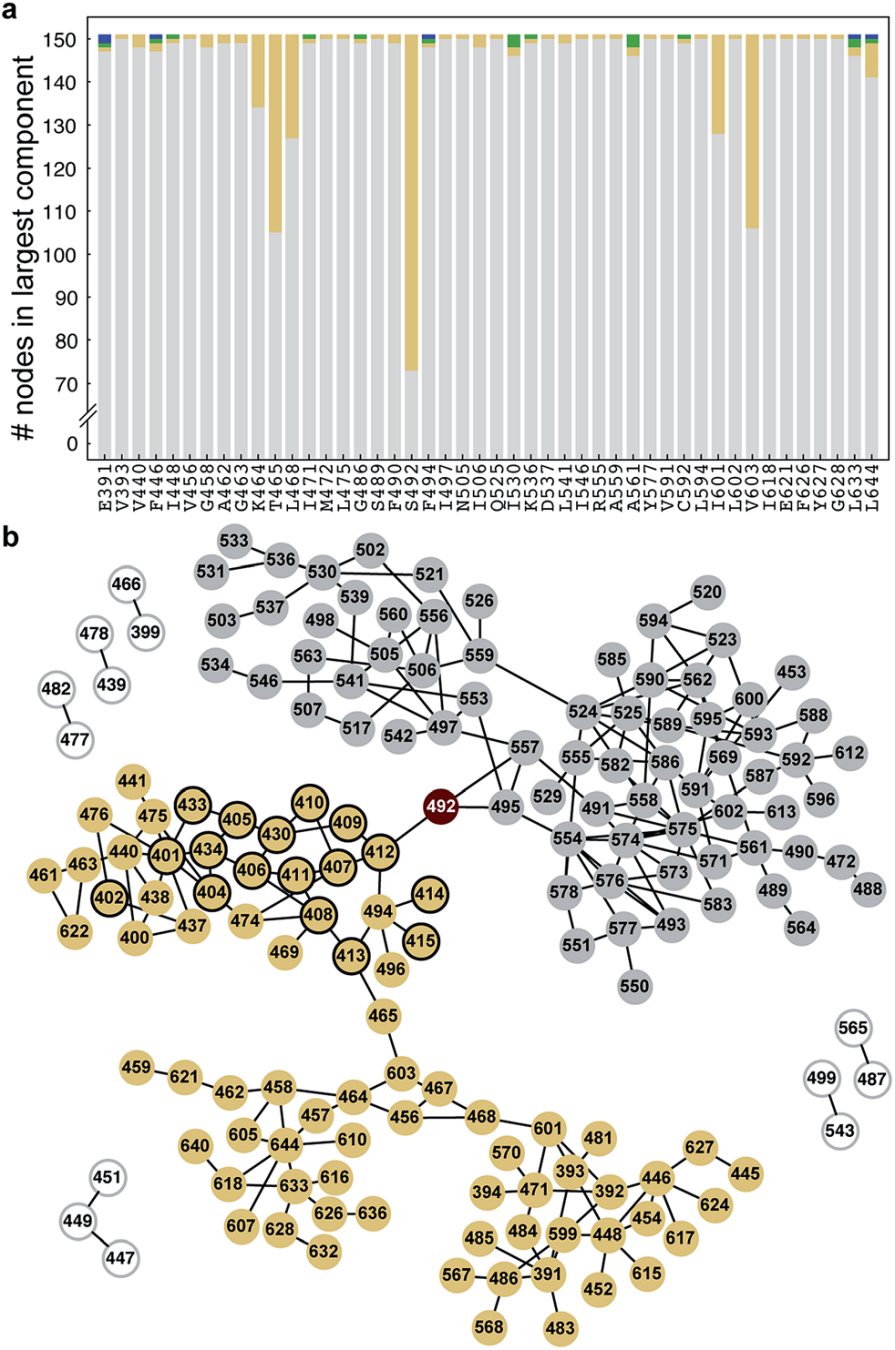
S492 is a critical node in ΔF508-NBD1. (a) Number of nodes in the largest component (*y*-axis) of the disconnected sub-graph after disconnecting the corresponding residue (*x*-axis). We consider residues that partition the sub-graph into connected components of ten or more residues as critical nodes. Different colors represent different components. (b) Network representation of the disconnected sub-graph. S492 forms a critical node that partitions the sub-graph into two connected components of nearly equal size (yellow and gray circles). Yellow nodes with black outline represent residues in the RI. Smaller components are represented as white circles.

We have previously reported that thermal fluctuations are increased in the SDR and dynamic coupling is lost between the F508 loop and the ATP-binding sub-domain upon deletion of F508, both of which are ameliorated upon deletion of the RI.^26^ We hypothesize that manipulating the coupling between the RI and the 508-loop and between RI and the SDR may ameliorate fluctuations and improve dynamic stability in these regions. In order to identify bottleneck residues that mediate the transfer of fluctuations in the RI across the domain, we conduct a network analysis of the correlated dynamics between residues in NBD1 (Methods). We conclude from this analysis that the residues K464, T465, L468, S492, I601, and V603 are critical nodes involved in the transfer of the thermal fluctuations from the RI to other regions of NBD1 *via* dynamic coupling (Fig. 2a). K464 and V603 mediate coupling with the β-strands forming the ATP-binding core sub-domain (Fig. S2,† red residues), L468 and L601 with the regulatory extension (RE) of NBD1 (Fig. S2,† blue residues), and T465 with both of these regions (Fig. 2b and S2†). However, S492 in β5 is the only residue mediating dynamic coupling between the RI and the 508-loop and SDR (Fig. 2b: yellow nodes; Fig. S2:† green residues). In addition, we note that S492 has been identified as the site of a rescue mutation for CFTR in an independent study.^18^ We conclude that S492 is a network bottleneck that dynamically couples the RI with these regions in NBD1.

### ΔI507-NBD1 network bottlenecks reside in regions essential for CFTR maturation and function

**Fig. 3:**
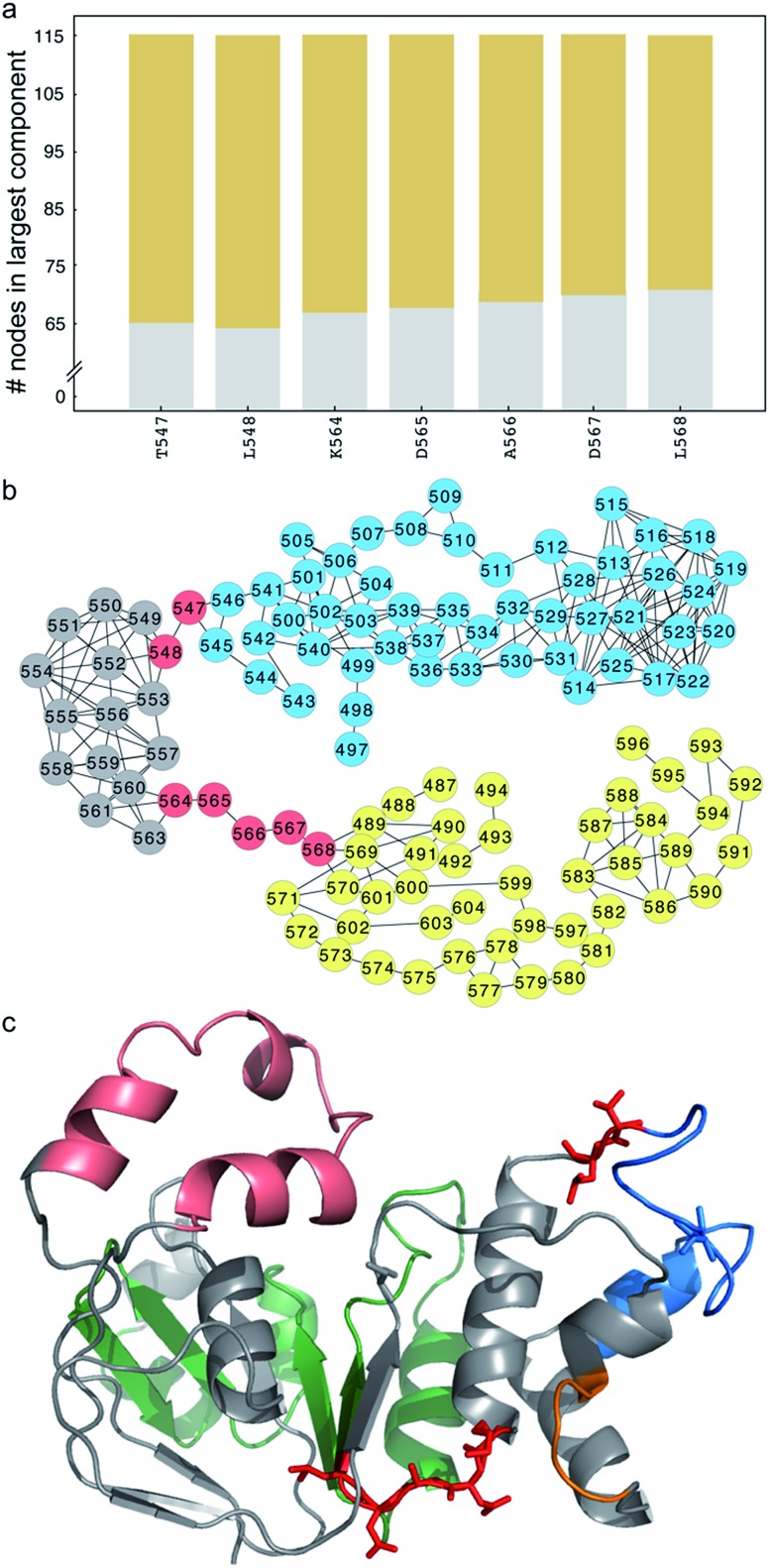
Critical nodes connect the SDR and ATP-binding subdomain in ΔI507-NBD1. (a) Number of nodes in the largest component (*y*-axis) of the disconnected sub-graph after disconnecting the corresponding residue (*x*-axis). We consider residues that partition the sub-graph into connected components of ten or more residues as critical nodes. Different colors represent different components. (b) Network representation of the disconnected sub-graph. Several residues form critical nodes that partition the sub-graph into two connected components of nearly equal size (yellow and blue circles; grey circles represent nodes that switch components depending on which critical node is removed). (c) Structure of NBD1 with critical nodes highlighted in red stick representation. Color-coded by sub-structure, the RI is pink, ATP-binding subdomain is green, SDR is blue, and F508 loop is orange. I539 (blue) and S492 (grey) are highlighted in stick representation.

Unlike ΔF508-CFTR, removal of the RI in NBD1 does not rescue ΔI507-CFTR, so we instead adopt the strategy of ameliorating the non-native coupling of the SDR and ATP-binding sub-domain. Using network analysis of correlated dynamics between ΔI507-NBD1 residues (Methods), we find that residues T547, L548, K564, D565, A566, D567, and L568 are critical nodes in the cross-domain coupling of dynamics in ΔI507-NBD1, and also that each of these critical nodes is central to coupling between the SDR and the ATP-binding sub-domain (Fig. 3a and b). However, residues T547 and L548 reside directly adjacent to or within the signature motif responsible for ATP binding,^35^ and residues D565, A566, and D567 comprise the di-acidic exit code recognized by coat complex II (COPII) for transport of CFTR from the endoplasmic reticulum to the membrane,^36,37^ with K564 and L568 directly adjacent (Fig. 3c). Although several reversion mutations exist in the signature motif,^24,38,39^ T547 and L548 appear to be in position to participate in the binding of ATP (Fig. 3c), one of the major contributing factors to CFTR stability.^40,41^ Using computational methods, we find potential stabilizing mutations at some of these sites (although only marginally stabilizing, ΔΔ*G* ∼1–2 kcal mol^–1^), but experiments confirm that these mutations result in lack of mature CFTR (Fig. S3†), reflecting the crucial nature of these conserved residues to full-length CFTR in the cell. While we therefore cannot utilize mutagenesis at these positions to rescue ΔI507-CFTR, we demonstrate that these important sites are hubs of coupled dynamics networks capable of affecting domain dynamics.

### S492P substitution restores dynamic stability and function to ΔF508-CFTR

**Fig. 4:**
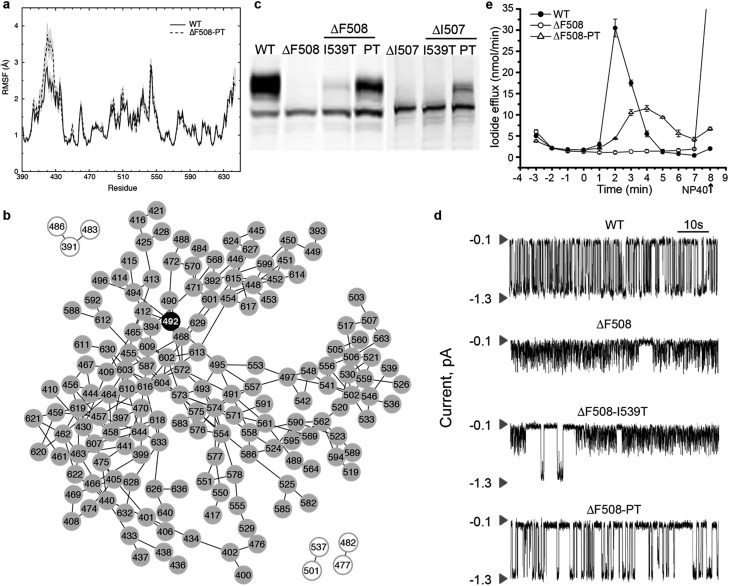
Recovery of maturation and functional regulation in mutant CFTR. (a) Root mean square fluctuations (RMSF) of residues in WT NBD1 compared with S492P substitution in the context of ΔF508-I539T-NBD1 and ΔI507-I539T-NBD1 denote stabilization of NBD1 upon mutation of S492. (b) Network representation of the subgraph from ΔF508-PT shows that the effect of RI is no longer propagated *via* P492 or any other nodes, but rather distributed across multiple nodes, indicating dissipation of thermal fluctuations upon mutation and consequent stabilization of the domain. (c) Western blot analysis of lysates from HEK293 cells transiently expressing WT or mutant CFTR confirms expression of all forms but maturation of only ΔF508-PT and ΔI507-PT CFTR. (d) Single channel recordings of wild type CFTR and mutant constructs at 35 °C in symmetrical salt solution (300 mM Cl^–^) under voltage-clamp at –75 mV. The upper arrow in each trace represents the closed state while the lower arrow is the open state of a single CFTR channel. We find partial recovery of WT function in only ΔF508-PT CFTR. Traces are plotted from data previously published^17^ (e) Iodide efflux measurements (Methods) in BHK cells stably expressing wild-type CFTR (solid circles), ΔF508-PT CFTR (open circles) or ΔF508-CFTR (solid triangles). Stimulation cocktail is added at time 0 to activate iodide efflux through CFTR channels. The values represent the mean ± standard deviation of the amount of iodide released from the cells during a one minute interval (*n* = 3). Efflux buffer containing 0.1% NP40 is added at the end of each assay (arrow) to release remaining iodide. We find that ΔF508-PT CFTR partially recovers WT activity as compared to ΔF508-CFTR.

To confirm that ΔF508-NBD1 dynamics can be improved by restoring coupling between the F508 loop and the ATP-binding sub-domain, we perform computational mutagenesis of S492 in the ΔF508 background. In an alignment of NBD1 with NBD2 and nucleotide-binding domains from many other ABC proteins, we find that the position corresponding to S492 contains exclusively either serine or proline (Fig. S4†).^18^ We therefore test the effect of a proline substitution at this position in NBD1. We perform DMD simulations and RMSF analysis of the NBD1 variant ΔF508-S492P in the context of I539T, a previously identified maturation reversion mutation^24,25^ (ΔF508-PT, generated using Medusa^15,42,43^). We find that ΔF508-PT NBD1 exhibits a thermal fluctuation profile comparable to that of wild type NBD1, suggesting potential thermal rescue of ΔF508-CFTR (Fig. 4a, in comparison to Fig. 1a). We demonstrate that this rescue of dynamics is due to the S492P substitution, as I539T mutation alone does not return wild type dynamics to ΔF508-NBD1 (Fig. S5†). Notably, the network of correlated dynamics in ΔF508-PT does not contain any bottlenecks; no single residue is so crucial to the dynamics that without it the network splits into non-communicating pieces (Fig. 4b). Such radical topological rearrangement could be a consequence of decreased fluctuations in the RI due to strengthened dynamic coupling. Alternately, in ΔF508-PT NBD1 the fluctuations originating from the RI might dissipate throughout the domain such that they are not channeled towards the 508-loop or the SDR.

To determine whether ΔF508-PT CFTR is functionally rescued at physiologically relevant temperatures, we perform experimental characterization of the ΔF508-PT variant of CFTR. As predicted from our simulations, Western blot analysis of lysates from HEK293 cells transiently expressing wild type and mutant (ΔF508, ΔF508-PT) CFTR reveals that maturation of ΔF508-PT is improved to a level similar to that of wild type CFTR (Fig. 4c). Using single channel recordings, we found in a previously published study^18^ that ΔF508-PT CFTR is functional at 35 °C while ΔF508-I539T is not, suggesting that the S492P substitution not only improves maturation but also restores function to ΔF508-CFTR (Fig. 4d). Furthermore, this result indicates that it is the dynamic stability of the protein, not evasion of the cell's quality control machinery such as attained by the I539T substitution, that guarantees CFTR function. These single channel tracings reveal that the fast, flickering gating mode characteristic of ΔF508-CFTR is interrupted only rarely by normal full conductance gating transitions in the I539T variant, whereas normal gating transitions are the dominant mode in ΔF508-PT CFTR. When assessed near human physiological temperature, ΔF508-PT features a level of channel activity that provides conductance similar to wild type CFTR (Fig. 4d). In parallel, we monitor iodide efflux from BHK cells stably expressing wild type, ΔF508, or ΔF508-PT CFTR (Fig. 4e). Together, the relative single channel properties of ΔF508-PT and wild type CFTR (Fig. 4d) and iodide efflux behavior (Fig. 4e) provide substantial evidence that ΔF508-PT CFTR trafficked to the plasma membrane is functional, and that the S492P mutation identified with our graph theoretical approach successfully rescues ΔF508-CFTR.

### S492P substitution restores partial wild-type function in ΔI507-CFTR

Because of the similarities in increased fluctuations and non-native coupling of the α-subdomain between ΔI507-NBD1 and ΔF508-NBD1, we hypothesize that the S492P mutation may have a similar rescue effect in ΔI507-NBD1. In simulations of ΔI507-PT NBD1, we find that fluctuations are ameliorated in comparison with ΔI507-NBD1, returning to a wild type-like profile (Fig. 4a). Notably, this dynamic stabilization results in wild type-like maturation (Fig. 4c). These results suggest that stiffening the RI-SDR bridge with a proline mutation at position 492 prevents the transfer of correlated dynamics from the SDR to both the RI and the ATP-binding subdomain, alleviating the changes in coupled motions and rescuing the mutant.

## Discussion

Allostery in proteins is viewed as an effect that propagates tens of angstroms across the structure, an action caused by a perturbation at a distant site. Described 50 years ago, this phenomenon is ubiquitously observed in various biological processes, from gene regulation to protein biogenesis. However, the mechanism of coupled dynamics transfer (caused by ligand binding, for example) over large distances (>10 Å, spanning multiple bond lengths) remains unknown, and determining this mechanism is a fundamental pursuit of structural biology.^4^ We utilize graph theoretical methods to determine the coupling between regions that mediates dynamics transfer and apply our methodology to rescue cystic fibrosis-causing mutants of the protein CFTR. We present two major advances: (i) development of a generally-applicable method to determine inter-residue interactions mediating *trans*-domain coupled dynamics in proteins (Fig. S6†) and (ii) rescue of CF-causing mutant forms of the CFTR chloride ion channel. Using our methodology, we not only detect the networks of inter-residue interactions that mediate dynamic coupling of distal protein regions, but also redesign these networks, demonstrating the ability to modulate protein dynamics to achieve specific goals. The applicability of our methodology is not limited to CFTR, but can be extended to any macromolecule of interest. Since the method involves representation of a protein using a generic mathematical construct, a graph, the concept is applicable to all macromolecules that are known to feature coupled dynamics, including RNA.

During the preparation of this work, Roy and Post^44^ have employed a similar method to examine the long-distance effects of drug binding in viral capsids. However, that work studies only local networks, defining edges only between those residues featuring separation less than 14 Å, regardless of the correlation between those residues. Our method of including all pairwise edges and culling edges featuring a correlation less than a cutoff value gives the dual advantage of increased network coverage with the inclusion of distal correlations and faster computation of shortest path, since weak connections are eliminated and are not included in calculations of shortest path.

In the present study, we apply our computational methodology to study dynamic coupling within NBD1 of CFTR. We identify hubs in NBD1 that mediate dynamic coupling between various sites across the protein (Fig. S2†), and find that the residue S492 alone mediates dynamic coupling between the dynamic RI region and the F508 loop where disease-relevant deletion occurs, making this residue a hot spot for rational design *via* mutagenesis. We further find that mutation of S492 to proline (an evolutionarily-conserved amino acid in this position among NBDs) successfully rescues ΔF508- and ΔI507-CFTR. To evaluate whether the rescue of CFTR upon S492P mutation is due simply to thermodynamic stabilization, we calculate the ΔΔ*G* of mutation at this position using Eris (Methods).^42,43^ Intriguingly, we find that the thermodynamic stability of ΔF508-NBD1 is not improved by proline substitution, nor by any amino acid substitution at this position, emphasizing the role of dynamic coupling within NBD1 in CFTR structural stability (Fig. S7†). The conservation of S492 in CFTR among various species further indicates an evolutionary basis for an dynamic coupling mechanism of regulation of NBD1 dynamics (Fig. S4†). Furthermore, we find both computationally and experimentally that mutagenesis of S492 in the context of I539T (referred to as ΔXXXX-PT), a previously identified maturation reverting mutation,^24^ significantly improves the dynamic stability, maturation, and function of CFTR deletion mutants. The biological significance of the combined I539T and S492P substitutions is also apparent in nature. For instance, some non-mammalian (*e.g. Xenopus laevis* and *Squalus acanthias*) CFTR orthologs naturally possess T539 and P492, and the ΔF508 mutation does not compromise their folding and maturation (Fig. S4 and S8†). Notably, mammalian species (*e.g. Equus caballus* and *Ovis aries*) do not contain these variations, and their folding and maturation is affected by F508 deletion (Fig. S4 and S8†).

In order to demonstrate the importance of dynamic behavior in the *trans*-domain “communication” between various regions, for contrast we perform a similar analysis of the static structure of human NBD1, using the number of contacts made between each pair of residues as the weight of each edge in the graph. In the graph of the static system, S492 does not appear in any of the paths between the RI and the F508 loop, further supporting the idea that an understanding of protein dynamics is critical to rational manipulation of proteins.

In the case of CF, the main cause of disease (approximately 90% of cases) is the deletion of F508 from NBD1 of CFTR.^17^ The deletion of I507, the adjacent residue, also results in disease.^45^ We find that deletion of these residues from the F508 loop substructure leads to non-native dynamic coupling interactions within NBD1. In ΔF508-NBD1, the deletion mutation causes a perturbation that decouples the RI from the rest of NBD1, and the loss of coupling with the RI results in non-native coupling within the α-subdomain and the ATP-binding domain. In ΔI507-CFTR, the deletion of I507 changes the interactions of the mutation site with the SDR, from which perturbations affect coupling throughout the domain. This difference explains why the removal of RI rescues ΔF508-CFTR, but does not affect ΔI507-CFTR (Fig. S9†). However, in both CFTR constructs, the perturbed SDR plays a major role in mediating transfer of aberrant dynamics from the mutation site. The substitution of a proline for a serine at position 492 in NBD1 stiffens the protein backbone and inhibits fluctuations, inhibiting the coupling between the SDR and both the RI and the ATP-binding sub-domain, interrupting the transfer of non-native dynamics and rescuing both deletion mutants. Notably, the phenomenon of lost native coupling in the ATP-binding sub-domain of ΔI507-NBD1, but not in ΔF508-NBD1, can potentially explain why the latter, but not the former, mutant can be rescued by decreasing the temperature from 37 °C to 27 °C (Fig. S9†); because the binding of ATP to the NBDs is known to be a significant stabilizing factor in CFTR,^40,41^ the loss of coupling between the different areas of the ATP-binding subdomain could interfere with the binding of this important ligand.

Given these findings and conclusions, we are faced with the question: if the S492P mutation rescues both ΔF508- and ΔI507-CFTR, why do we not identify S492 in our computational analysis of the ΔI507-NBD1 dynamic network? We note that coupled motions are markedly lower in ΔI507-NBD1 as opposed to ΔF508, as evidenced by the much lower correlation coefficient cutoff in our networks (0.50 for ΔF508, 0.32 for ΔI507). We hypothesize that the low level of correlation in the ΔI507-NBD1 domain and sharp transition in node inclusion (Fig. S1†) make the choice of network cutoff much more critical to network properties. We find that S492 is indeed located in the bottleneck region, in fact correlated (correlation coefficient 0.30, as compared to the cutoff 0.32) with one of the identified bottleneck residues, L568. We note that in using our method, an expert researcher could manually fine-tune the correlation coefficient cutoff based on prior knowledge or on regions or residues of interest. Manual intervention may be particularly useful in systems such as ΔI507-NBD1, where the sharp transition in network composition with correlation coefficient cutoff makes choice of this parameter particularly critical.

The sensitivity of the ΔI507-NBD1 system leads us to emphasize that our method serves as a guide to narrow the wide field of possible rescue mutations, highlighting residues with potential influence over protein dynamics, and that not all identified residues will be the site of viable rescue mutations, nor that all rescue mutation sites will be identified. The computational methods that we employ necessarily are molecular in scale, and cannot integrate all cellular processes that influence the expression, maturation, and interactions of particular mutants. Also due to molecular detail, our computational method is only applicable in cases where the structure of the protein or protein domain of interest has been solved to high resolution (<4 Å). Lower resolution structures would require additional computational methods to model atomic resolution before our approach could be applied. Finally, the structure must be of a size that is practical for extensive dynamic sampling during molecular simulations.

## Methods

### NBD1 models and discrete molecular dynamics simulations

Crystal structures exist of wild type NBD1 (PDB ID: 2BBO) and ΔF508-NBD1 (PDB ID: ; 2BBT), but because of the inherent flexibility of the RI this region is not resolved in either structure. We reconstruct the missing residues in the RI *ab initio* using discrete molecular dynamics (DMD) simulations^30^ with the Medusa force field.^15^ We perform multiple iterations of replica exchange simulation and energetic minimization using Chiron^46^ to obtain final minimized models of full-length wild type and ΔF508-NBD1. No structure of ΔI507-NBD1 is currently available, therefore starting from our minimized model of full-length wild type NBD1, we manually delete the I507 residue and reseal the protein backbone using DMD simulations. We reseal the protein backbone by imposing peptide-bonding constraints on the cleaved ends until the backbone is once again intact. We hold the majority of the protein static during this process, allowing only three residues on either side of the deletion to move freely and reseal the gap caused by deletion. From these three initial full-length models (wild type, ΔF508, and ΔI507), we utilize the Eris suite^42,43^ to create S492P/I539T mutants of each construct. We perform energy minimization of all constructs using Chiron.

We perform long timescale single-temperature DMD simulations using the final minimized models of full-length NBD1. We perform 10 randomized simulations for each construct at a temperature of 0.4 kcal mol^–1^ *k*_B_^–1^, with each individual simulation having a length of 10^6^ DMD time steps (approximately 50 ns), summing to a total of approximately 500 ns for each construct. The results that we present here are averaged over all simulations for each construct, unless otherwise specified.

### Construction of dynamic networks and determination of optimal paths

We utilize the resulting simulation trajectories to determine dynamic coupling between the various regions of NBD1 by computing correlation coefficients of motion between all residues.^32^ We represent the pairwise correlation map as a complete weighted graph G(N,E), with the C_α_ atoms in NBD1 representing the nodes (N) of the graph and the edges (E) being the connections between these nodes. The edge weight *E*_*ij*_ between any two nodes *i* and *j* is the correlation coefficient *C*_*ij*_ between the corresponding pair of residues.

To determine optimal paths of dynamics transfer through coupled residues, we use the same network with edge weights of *E*_*ij*_ = 1 – |*C*_*ij*_| and apply Dijkstra's algorithm.^47^ Since dynamic coupling is mediated by physical interactions, we reduce the complete graph by removing edges between nodes representing residues that are not in contact. We consider two residues to be in contact if their C_β_ atoms (C_α_ for Glycine) are within 7.5 Å of one another. Our aim is to determine the transfer of correlated motion across the protein. Hence, we emphasize the effect of local interactions by retaining only those edges in which the participating nodes feature a contact frequency (*ω*_c_) of at least 0.5 over the simulation.

### Flexible backbone redesign

We perform iterative flexible backbone redesign and structural relaxation using the Medusa suite^15^ in order to ensure optimum backbone configuration for computational mutagenesis of human NBD. We perform 15 randomly-seeded iterations of backbone redesign Monte Carlo simulations with 20 Monte Carlo iterations per replicate. We select the lowest energy structure from each iteration to use as the starting point for the next iteration. Finally, we choose the structure with lowest energy and smallest Kabsch root mean square deviation (KRMSD) from the initial structure for fixed-backbone mutation analysis using the Eris suite.^42,43^

### CFTR construction and expression

We express human CFTR cDNAs encoding wild type or mutant proteins transiently in HEK 293 cells or stably in BHK-21 cells, with pcDNA3 or pNUT vectors, respectively, as previously described.^28^ We use the QuickExchange protocol (Stratagene) to generate mutant CFTR constructs in pcDNA3 and pNUT vectors from human WT CFTR cDNA, and confirm sequences by automated DNA sequencing (UNC-CH Genome Analysis Facility). We carry out transfection using jetPEI transfection reagent (Fermentas, Glen Burnie, MD) according to the manufacturer's instructions. For stable cell line establishment, we select and maintain BHK cells expressing CFTR in methotrexate-containing media as previously described.^48^

### Western blotting

We harvest HEK or BHK cells overexpressing CFTR in radioimmunoprecipitation assay (RIPA) buffer without SDS (50 mM Tris, 150 mM NaCl, 1% Triton X-100, 1% deoxycholate, pH 7.4) plus protease inhibitor cocktail (1 μg ml^–1^ leupeptin, 2 μg ml^–1^ aprotinin, 3.57 μg ml^–1^ E64, 156.6 μg ml^–1^ benzamidine and 2 mM Pefablock). We subject equal amounts of proteins in SDS-PAGE sample buffer to 7.5% SDS-PAGE and Western blot analysis with mAb596 in order to determine CFTR expression and maturation.^49^

### Membrane isolation

We harvest BHK or HEK 293 cells expressing CFTR or its variants by scraping, and then homogenize the cells on ice in 10 mM Hepes, pH 7.2, 1 mM EDTA (ethylenediaminetetraacetic acid) containing a protease inhibitor cocktail (benzamidine at 120 μg ml^–1^, E64 at 3.5 μg ml^–1^, aprotinin at 2 μg ml^–1^, leupeptin at 1 μg ml^–1^ and Pefablock at 50 μg ml^–1^). We centrifuge the resulting samples at 600 g for 15 minutes to remove nuclei and undisrupted cells, followed by centrifugation at 100 000*g* for 60 minutes to pellet the membranes, which we then resuspend in phosphorylation buffer (10 mM Hepes, pH 7.2, containing 0.5 mM EGTA (ethylene glycol bis(β-aminoethyl ether), *N*,*N*′-tetraacetic acid), 2 mM MgCl_2_, and 250 mM sucrose). We utilize brief (3 × 20 s) bath sonications to generate vesicles of uniform size. For single-channel recordings, we phosphorylate membrane vesicles by incubating with 50 nM PKA catalytic subunit (Promega) and 2 mM Na_2_ATP (Sigma) in phosphorylation buffer for 20 min at +4 °C. We then aliquot the membranes and store at –80 °C for later use.

### Single-channel measurements

To prepare planar lipid bilayers, we drill a 0.2 mm hole in a Teflon cup and paint the hole with a phospholipid solution containing a 3 : 1 mixture of 1-palmitoyl-2-oleoyl-*sn-glycero*-3-phosphoethanolamine and 1-palmitoyl-2-oleoyl-*sn-glycero*-3-phosphoserine (Avanti Polar Lipids) in *n*-decane. The lipid bilayer separates 1 ml of solution in the Teflon cup (*cis* side) from 5 ml of solution in the outer glass chamber (*trans* side). Both chambers are magnetically stirred and thermally insulated. We utilize a temperature control system (TC2BIP, Cell Micro Controls).

We transfer CFTR ion channels into the pre-formed lipid bilayer by spontaneous fusion of membrane vesicles containing the CFTR variants. To maintain uniform orientation and functional activity of the CFTR channels transferred into the bilayer, we add 2 mM ATP, 50 nM PKA, and membrane vesicles into the *cis* compartment only. We perform all measurements in symmetrical salt solution (300 mM Tris–HCl, pH 7.2, 3 mM MgCl_2_ and 1 mM EGTA) under voltage-clamp conditions using an Axopatch 200B amplifier. We maintain a membrane voltage potential of –75 mV, the difference between *cis* and *trans* (ground) compartments. We analyze the resulting data as previously described.^50^

### Iodide efflux assay

We grow BHK cells stably expressing wild type and mutant CFTR to ∼100% confluence in six-well plates and incubate in an iodide loading buffer (136 mM NaI, 3 mM KNO_3_, 2 mM Ca(NO_3_)_2_, 11 mM glucose, and 20 mM Hepes, pH 7.4) for one hour at room temperature. We rinse the cells with iodide-free efflux buffer (which is the same as the loading buffer except that NaI is replaced by NaNO_3_) to remove extracellular iodide. We collect samples by completely replacing the efflux buffer (1 ml volume) with fresh solution at one-minute intervals. We use results from the first four samples to establish a baseline. We measure iodide efflux upon stimulation with PKA agonists (10 μM forskolin, 100 μM dibutyl-cAMP and 1 mM 3-isobutyl-1-methylxanthine) using an iodide-selective electrode LIS-1461CM (Lazar Res. Lab., Inc.), as previously described.^48^

## Conclusions

We demonstrate here that NBD1 of CFTR, for which *trans*-domain coupled dynamics was not previously described, features patterns of correlated motions that form a network throughout the domain and allow structural fluctuations to be transferred to distal sites through dynamic coupling, supporting the general notion that all folded proteins are to some extent allosteric in nature.^3^ Moreover, we show that these networks can be rationally redesigned to affect a desired outcome. Identifying the residues involved in *trans*-domain dynamics can provide a map to greatly improve the stability and/or modulate the function of enzymes for numerous biotechnological applications, as well as contribute to our understanding of the many human diseases caused by protein dysfunction. Determining dynamic coupling networks and hot spots is also of practical importance in rational drug design.^3^ Conventionally, structure-based virtual screening utilizes experimentally-validated binding sites, such as an enzyme catalytic site or a ligand-bound pocket, but the determination of pre-existing dynamic communication pathways has great potential for identifying novel “druggable” sites that can be targeted to modulate protein function in human diseases. Thus, beyond the significant advance in understanding the fundamental defect in cystic fibrosis, our approach has broad application in the elucidation of mechanisms of protein function and dysfunction in disease.
