# Supplementary material for: Rational coupled dynamics network manipulation rescues disease-relevant mutant cystic fibrosis transmembrane conductance regulator
Source: Chem Sci. 2014 Nov 20;6(2):1237–46. doi: 10.1039/c4sc01320d (PMC4324596; doi:10.1039/c4sc01320d)
Supplement: Supplementary file 1 [file SC-006-C4SC01320D-s001.pdf]

This journal is © The Royal Society of Chemistry 2014

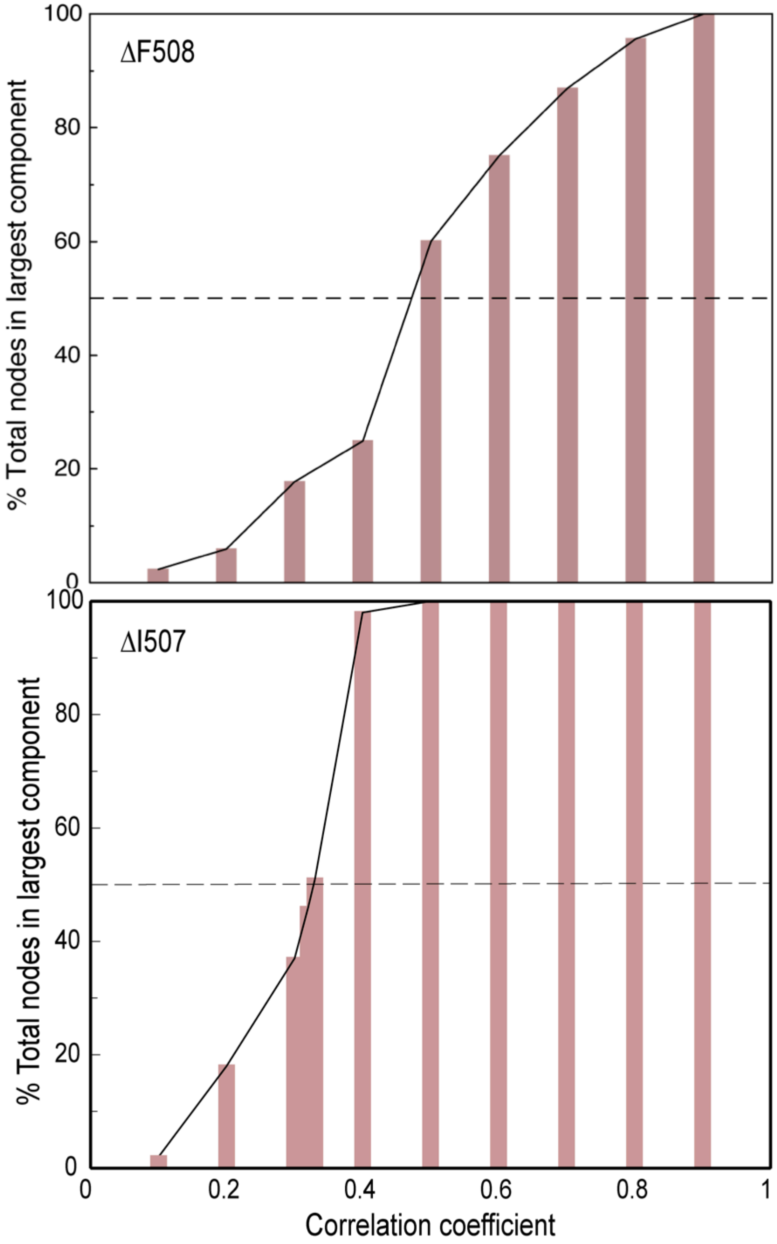

Figure S1. Choice of cutoffs for disconnected sub-graphs. Percentage of the total number of nodes in the graph (y-axis) as a function of correlation coefficient (x-axis) for the ΔF508 (top) and ΔI507 (bottom) networks. The correlation coefficient at which at least half the total number of nodes in the graph (half of the protein residues) reside in the largest connected component is chosen as a cutoff in generating the disconnected sub-graph. The dashed line represents half of the maximum number of nodes in the graph.

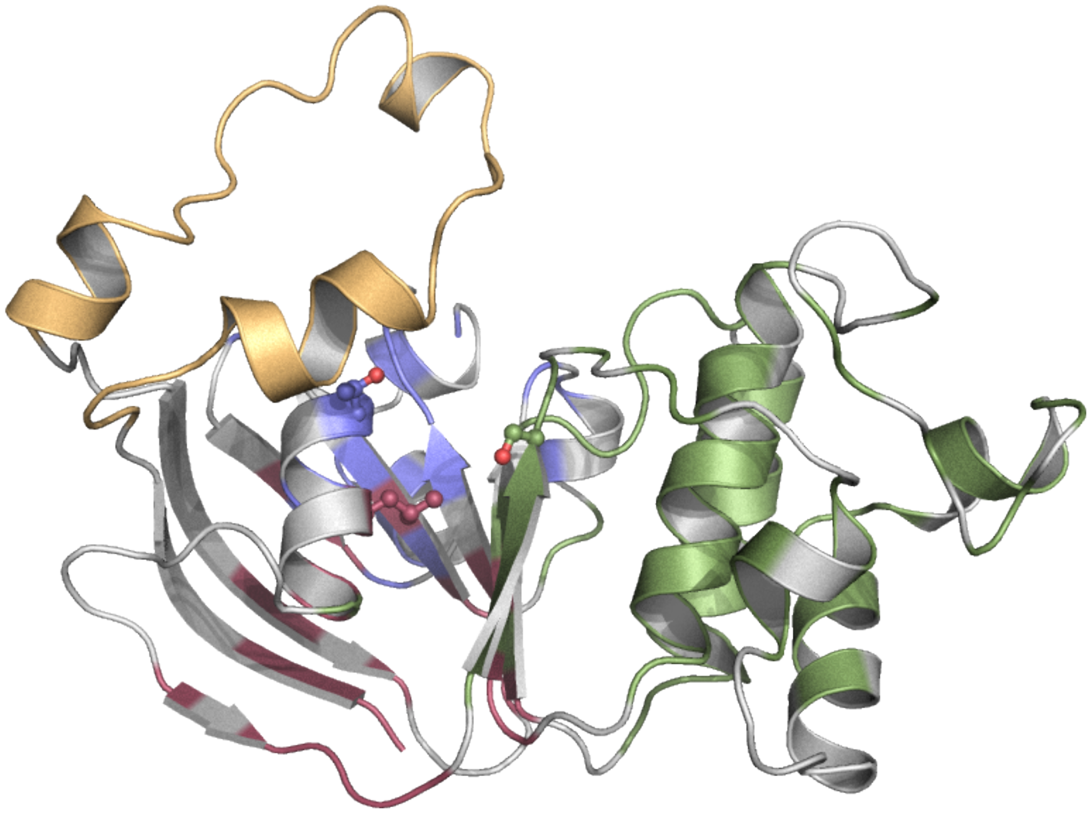

Figure S2. Regions in NBD1 affected by critical nodes. Three critical nodes – S492, T465, and L468 – are shown in ball-and-stick representation. S492 influences residues shown in green, T465 influences those shown in blue and red, and L468 influences those in red. Other critical nodes affect subsets of the residues colored red, green, and blue.

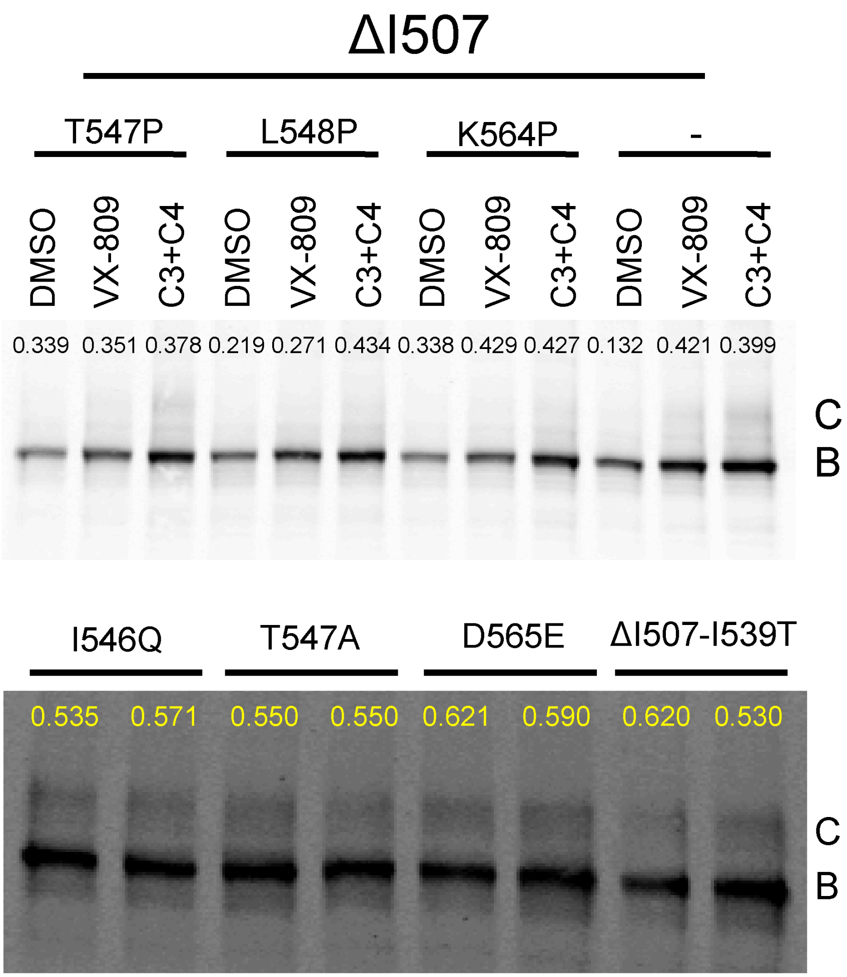

Figure S3. Mutations to critical nodes affect critical maturation pathways in ΔI507-CFTR. HEK cells transiently transfected with ΔI507-CFTR with or without additional mutations. We treat cells with indicated correctors 48 hours post-transfection, and grow at 27°C for an additional 24 hours before harvesting for western blot analysis (20 μg proteins/lane). Ratio of C-band to B-band is displayed below each lane.

Q07DV2	*Aotus nancymaae*	487-RISFCSQFSWI-497
Q00PJ2	*Atelerix albiventris*	487-RISFCSQFSWI-497
Q09YK5	*Ateles geoffroyi*	487-RISFCSQFSWI-497
P35071	*Bos taurus*	486-RISFCSQYSWI-496
Q2QLB4	*Callicebus moloch*	487-RISFCSQFSWI-497
Q2QLF9	*Callithrix jacchus*	487-RISFCSQFSWI-497
Q5U820	*Canis fimiliaris*	487-RISFCSQFSWI-497
Q2QLC5	*Carollia perspicillata*	487-RISFCSQFSWI-497
Q07E05	*Cavia porcellus*	487-RVSFCSQFSWI-497
Q2IBA1	*Cercopithecus aethiops*	487-RISFCSQFSWI-497
Q07DY5	*Colobus guereza*	487-RISFCSQFSWI-497
A0M8W1	*Danio rerio*	486-RISYSSQTAWI-496
Q07E42	*Dasypus novemcinctus*	487-RISFCSQFSWI-497
A4ZE37	*Dicentrarchus labrax*	486-RISFSPQTSWI-496
Q2QL74	*Didelphis marsupialis*	487-RISFCSQFSWI-497
Q2QLA3	*Equus caballus*	486-RISFCSQFSWI-496
A0M8T4	*Felis catus*	487-RISFCSQFSWI-497
A0M8U4	*Gallus gallus*	488-RISFSPQVSWI-498
Q2IBF6	*Gorilla gorilla*	487-RISFCSQFSWI-497
P13569	*Homo sapiens*	487-RISFCSQFSWI-497
Q07DX5	*Hylobates leucogenys*	487-RISFCSQFSWI-497
Q108U0	*Loxodonta africana*	487-RISFCSQFSWI-497
Q7JII8	*Macaca fascicularis*	487-RISFCSQFSWI-497
Q7JII7	*Macaca fuscata*	487-RISFCSQFSWI-497
Q9TUQ2	*Macaca memestrina*	487-RISFCSQFSWI-497
Q00553	*Macaca mulatta*	487-RISFCSQFSWI-497
A4D7T4	*Macropus eugenii*	487-RISFCSQFSWI-497
Q2QL83	*Microcebus murinus*	487-RISFCSQFSWI-497
Q09YJ4	*Muntiacus muntjak*	486-RISFCSQYSWI-496
Q07DW5	*Muntiacus reevesi*	486-RISFCSQYSWI-496
P26361	*Mus musculus*	487-RVSFCSQFSWI-497
Q07E16	*Mustela putorius*	487-RISFCSQFSWI-497
Q07DZ6	*Ornithorhynchus anatinus*	487-RISFSPQFSWI-497
Q00554	*Oryctolagus cuniculus*	487-RISFCSQFSWI-497
Q2QLH0	*Otolemus garnettii*	487-RISFCSQFSWI-497
Q00555	*Ovis aries*	486-RISFCSQYSWI-496
Q2QLE5	*Pan troglodytes*	487-RISFCSQFSWI-497
Q9TSP5	*Papio anubis*	487-RISFCSQFSWI-497
Q2IBE4	*Pongo abelii*	487-RISFCSQFSWI-497
P34158	*Rattus norvegicus*	487-RVSFSSQISWI-497
Q2IBB3	*Rhinolophus ferrumequinum*	487-RISFCSQFSWI-497
Q09YH0	*Saimiri boliviensis*	487-RISFCSQFSWI-497
Q98TY5	*Salmo salar*	486-RISFSPQTSWI-496
P26362	*Squalus acanthias*	488-RISYSPQVPWI-498
Q6PQZ2	*Sus scrofa*	487-RISFCSQFSWI-497
Q5D1Z7	*Trichosurus culpecula*	487-RISFCSQFSWI-497
P70034	*Xenopus laevis*	488-RISFSPQVSWI-498

Figure S4. Sequence conservation in CFTR from different species. Amino acid sequences of CFTR from different species from the Uniprot database. For clarity, an 11 residue window with S492 (or equivalent) as the center of the window is shown. The serine at position 492 (or equivalent) is conserved in other species (red) with a substitution only to proline (green).

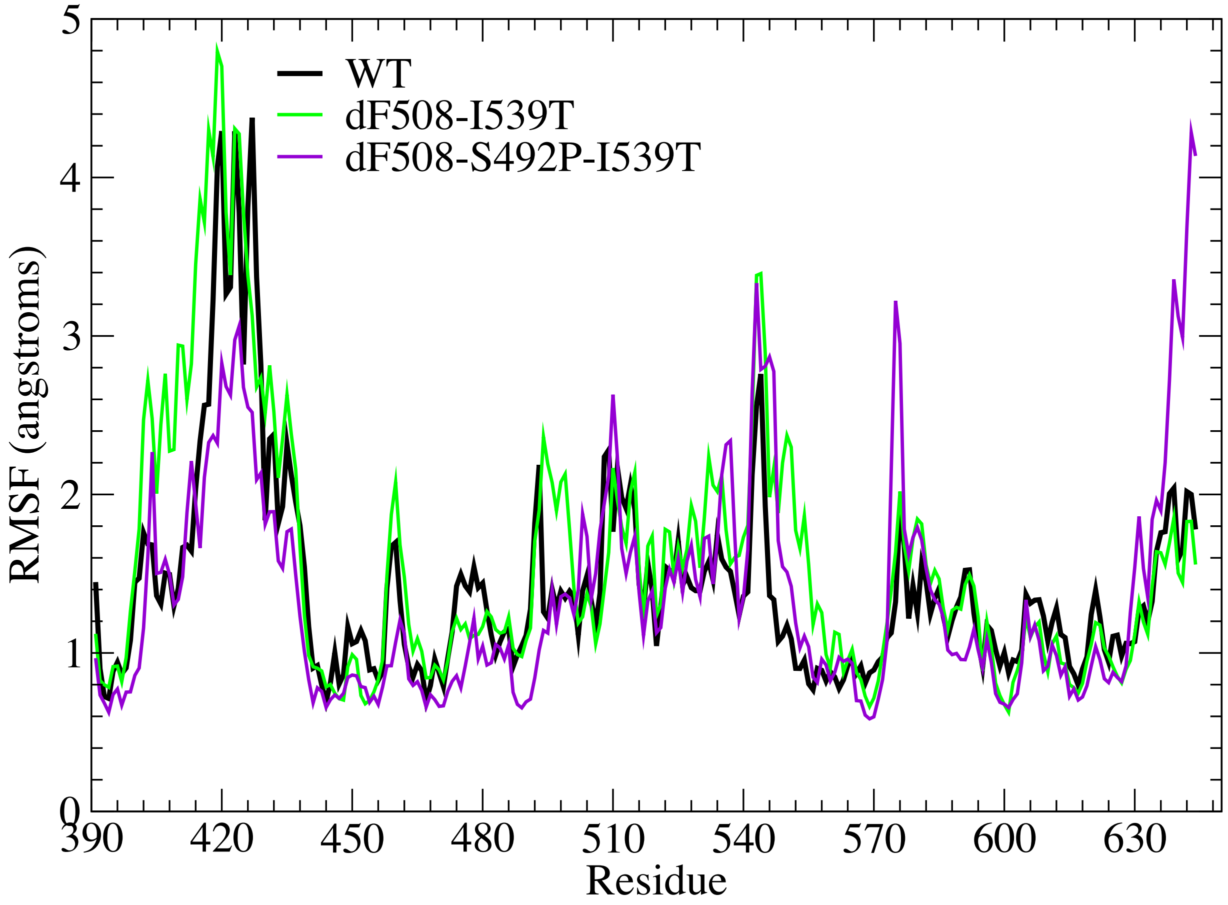

Figure S5. I539T mutation does not rescue NBD1 dynamics in ΔF508-CFTR. The I539T mutation, commonly used in the experimental setting to affect maturation of ΔF508-CFTR, does not return the profile of root mean square fluctuations (RMSF) (green) to wild type levels (black), especially in the dynamic regulatory insertion (RI) region, residues 404-435, and the critical central structure of the domain, residues 490-570. When combined with the S492P substitution (purple), these fluctuations are rescued.

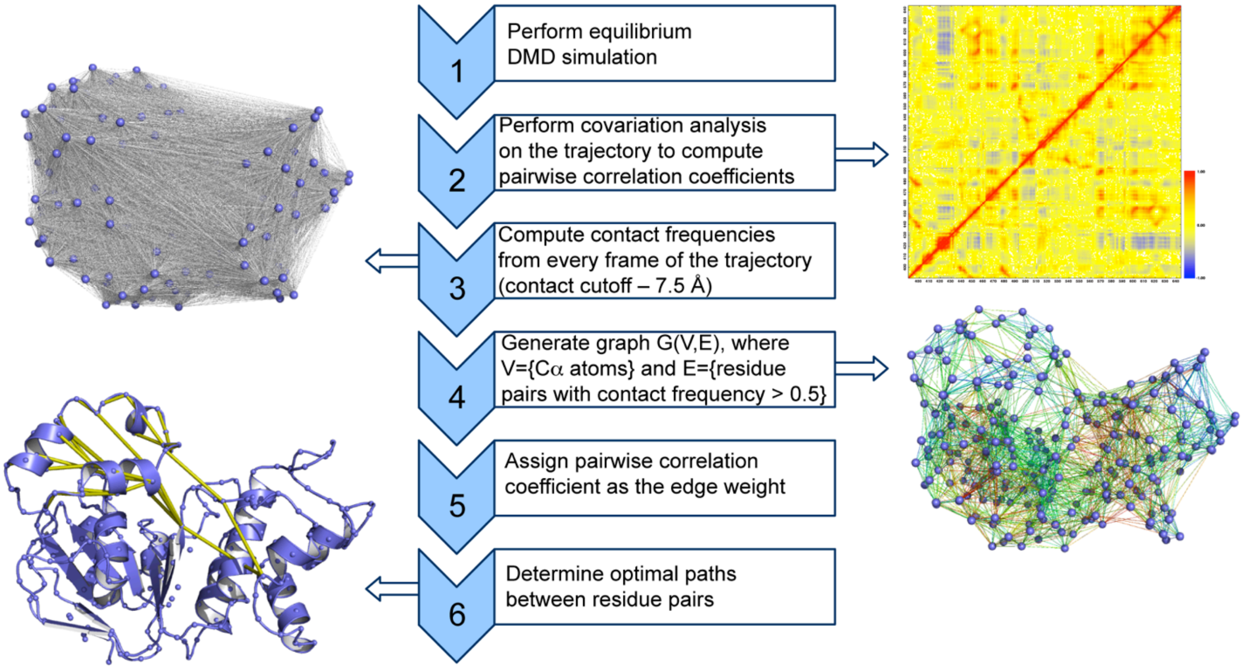

Figure S6. Computational methodology for determination of allosteric networks. The flow chart outlines the steps involved in determination of optimal paths between chosen sites in a given protein.

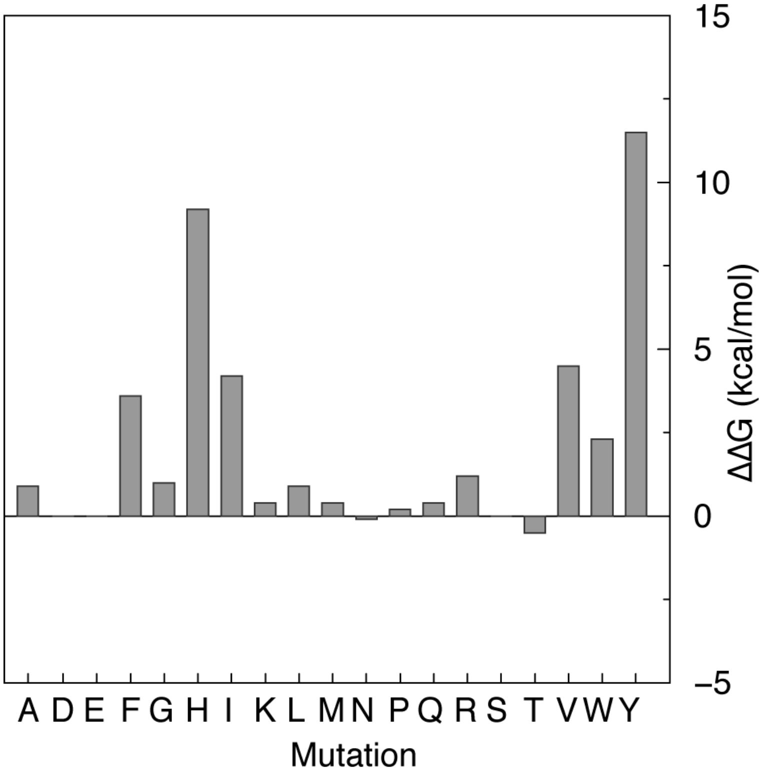

Figure S7. Exhaustive mutagenesis of S492 does not improve stability. Change in ΔG upon mutation of S492 to various amino acids. Negative values denote stabilizing mutations, while positive values denote destabilizing mutations. Bars represent ΔΔG values calculated from 500 independent iterations for every substitution.

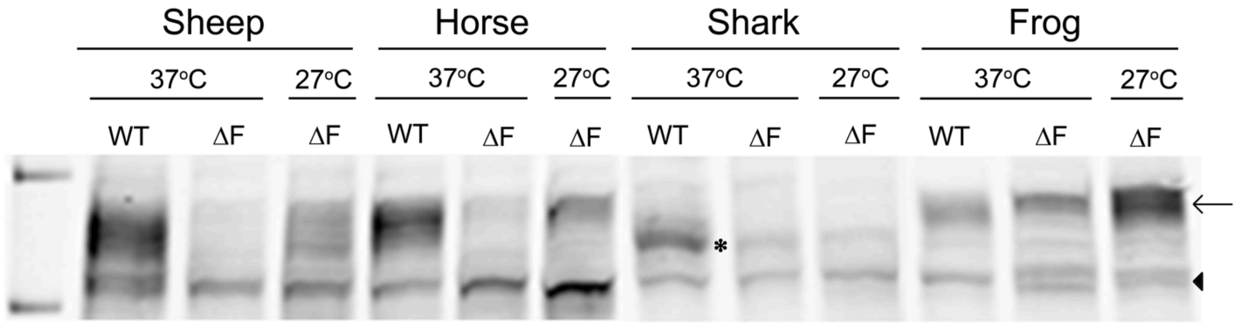

Figure S8. **Effect of F508 deletion on CFTR maturation in various species.** Western blots indicate the extent of maturation of WT and ΔF508-CFTRs from the species indicated in HEK-293 cells at 37°C or 27°C. The more rapidly migrating band (arrow) contains the core-glycosylated, endoplasmic reticulum localized form, and the more slowly migrating band contains CFTR with more complex oligosaccharides. The latter form of the shark protein (asterisk) features greater mobility than that of the other species due to the addition of one rather than two N-linked oligosaccharide chains. Like human ΔF508-CFTR, the two mammalian proteins mature at the lower but not at the higher temperature, whereas the non-mammalian orthologs mature at both temperatures.

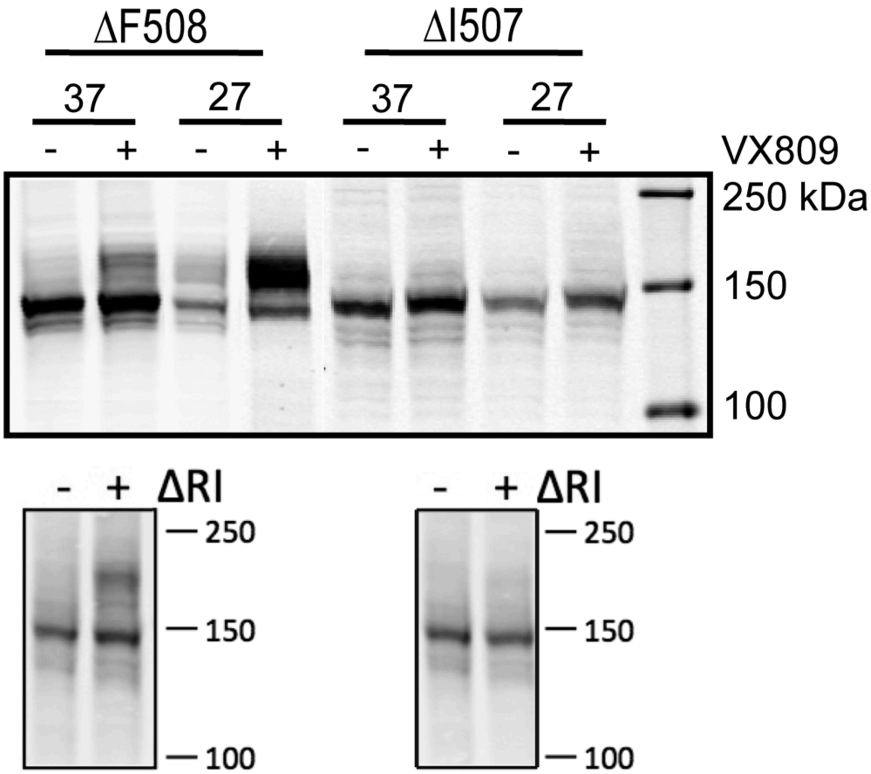

Figure S9. ΔI507-CFTR is not rescued by low temperature or VX809 corrector. While ΔF508-CFTR can be rescued by lowering temperature to 27 °C, treatment with VX809 corrector, or both, ΔI507-CFTR is not rescued by either of these treatments, alone or in combination.
